# The impact of the therapeutic alliance on treatment outcome in patients with dissociative disorders

**DOI:** 10.3402/ejpt.v5.22676

**Published:** 2014-03-06

**Authors:** Elisabeth Cronin, Bethany L. Brand, Jonathan F. Mattanah

**Affiliations:** Department of Psychology, Towson University, Towson, MD, USA

**Keywords:** Dissociative, alliance, trauma, treatment, PTSD

## Abstract

**Background:**

Research has shown that the therapeutic alliance plays an important role in enhancing treatment outcome among individuals with a variety of disorders, including posttraumatic stress disorder (PTSD). However, the therapeutic alliance and treatment outcome has not yet been studied in dissociative disorders (DD).

**Objectives:**

The current study sought to investigate the impact of alliance on treatment outcome for DD patients.

**Methods:**

Data from a naturalistic, longitudinal international treatment study of DD patients and their therapists were analyzed to determine if the alliance, as reported by patients and therapists, was associated with treatment outcome.

**Results:**

Patients with higher self-rated alliance had fewer symptoms of dissociation, PTSD, and general distress, as well as higher levels of therapist-rated adaptive functioning. Over time, self-rated alliance scores predicted better outcomes, after controlling for patient adaptive capacities including symptom management at the time when the alliance ratings were made. Patient-rated alliance was more strongly associated with outcome than therapist-rated alliance.

**Conclusion:**

Therapists who work with DD patients should understand the importance of the alliance on treatment outcome. These findings are consistent with previous literature demonstrating the importance of developing and maintaining a strong therapeutic alliance, although the effect sizes of individuals with DD were stronger than what has been found in many other patient groups. A greater understanding of the impact of the alliance in traumatized individuals may contribute to better outcomes for these individuals.

Dissociation is defined as “a disruption of and/or discontinuity in the normal integration of consciousness, memory, identity, emotion, perception, body representation, motor control, and behavior” (American Psychiatric Association [APA], 2013, p. 291). Dissociative disorders (DD) have an underlying neurobiological basis that includes excessive limbic inhibition and alterations involving the endogenous opioid system, among others (Brand, Lanius, Loewenstein, Vermetten, & Spiegel, 2012; Lanius et al., 2010). The APA categorizes dissociative identity disorder (DID) as the most severe of DDs, characterized by two or more personality states that show discontinuity in sense of self along with alterations in behaviors, memory, perception, cognition, and feelings, as well as amnesia for everyday events and/or traumatic experiences. DDNOS (Dissociative Disorder Not Otherwise Specified) occurs when patients experience a variety of dissociative symptoms that do not meet full criteria for any of the other DDs (e.g., DID symptoms with no amnesia) (APA, 2013).

Understanding, diagnosing, and treating DID is a serious problem for the mental health field. Despite being found in up to 5% of inpatients and 1% of the general population (International Society for the Study of Trauma and Dissociation [ISSTD], 2011), relatively few clinicians receive training in accurately diagnosing and treating DID, despite the existence of expert consensus guidelines and treatment recommendations (ISSTD, 2011; Brand et al., 2012). Even among clinicians trained in treating DID, treatment is challenging due to these patients’ wide range of chronic, severe symptoms and their difficulty trusting others, including mental health providers. Despite these difficulties, much progress has been made in the field to better understand DDs and their treatment (ISSTD, 2011).

DID and most cases of DDNOS are thought to be caused by ongoing, severe childhood traumas (e.g., Ross et al., 1991). Treatment of these disorders often takes place over a number of years, and the process is complicated by a number of commonly comorbid conditions and suicidal ideation (Rodewald, Wilhelm-Gössling, Emrich, Reddemann, & Gast, 2011; Foote, Smolin, Neft, & Lipschitz, 2008). Individuals with DID frequently meet criteria for a number of other axis I and II disorders. Rodewald et al. (2011) found that a sample of 66 female patients with DID/DDNOS had, on average, five comorbid disorders, the most common of which is posttraumatic stress disorder (PTSD).

Expert consensus guidelines suggest that DID therapy should be a trauma-informed, staged treatment, the main goals of which are to help the patient stabilize symptoms related to dissociation and PTSD; decrease self-destructive and suicidal behavior; develop awareness and cooperation with dissociated self-states; enhance affect awareness, tolerance and regulation; process and resolve traumatic experiences; and ultimately, develop a sense of being more internally whole and integrated (ISSTD, 2011; Brand et al., 2012). Experts have emphasized the importance of using the relationship between the client and therapist as a vehicle for helping clients understand and resolve their relational difficulties which relate to early trauma and attachment difficulties (Courtois & Ford, 2013; Dalenberg, 2004; Herman, 1997; Kluft, 1993a, 1993b). Despite the presence of established treatment guidelines, there still exists an urgent need for research about treatment outcome (Brand et al., 2013). The chronic suicidality and self-destructiveness that characterizes these disorders necessitates long-term treatment in most DID/DDNOS patients, yet such treatment is difficult to study, particularly with a randomized clinical trial design. The lack of funding for long-term treatments has further slowed treatment research.

The longitudinal, naturalistic treatment of patients with dissociative disorders (TOP DD) study conducted by Brand and colleagues (2009b, 2013) examined outcomes among DID/DDNOS patients treated by community clinicians over the course of 30 months of treatment. Results indicated patients improved across a range of symptoms. One important process variable that has not yet been examined in the TOP DD study, or in any study of DD patients, is the therapeutic alliance. This critical bond between therapist and client has been studied in almost all other patient populations, and has been consistently noted as an important factor in the therapeutic process. Using data from the TOP DD study, the current study examined the importance of therapeutic alliance in predicting improvement in outcomes over the course of treatment.

## The therapeutic alliance

The therapeutic alliance is commonly conceptualized as consisting of three main variables: an “affective bond,” and agreement on goals as well as tasks between the therapist and client (Martin, Garske, & Davis, 2000). In a recent qualitative study, clinicians also noted the importance of genuineness, flexibility, and ability to truly listen to a patient (Laska, Smith, Wislocki, Minami, & Wampold, 2013). Hilsenroth, Peters, and Ackerman (2004) found that early patient reports of alliance strength are predictive of strength of alliance at the end of treatment, a finding that indicates that the alliance is relatively steady over time.

Individuals who have been subjected to ongoing physical and sexual abuse are likely to encounter difficulties in forming trusting relationships later in life (Keller, Zoellner, & Feeny, 2010). Despite their trauma-based mistrust, many researchers have found a positive association between strong alliance and successful treatment outcome in survivors of abuse (Paivio & Bahr, 1998; Paivio & Patterson, 1999; Eltz, Shirk, & Sarlin, 1995; Cloitre, Stovall-McClough, Miranda, & Chemtob, 2004; Price, Hilsenroth, Callahan, Petretic-Jackson, & Bonge, 2004). Although survivors of abuse and trauma tend to have more difficulty forming and maintaining healthy relationships, researchers have found that these individuals are capable of forming strong therapeutic alliances (Keller et al., 2010; Price et al., 2004). Price et al. (2004) studied adult survivors of childhood sexual abuse (CSA), a type of abuse that the majority of DID patients report having experienced (e.g., Brand et al., 2009b). Price and colleagues found comparably high levels of therapeutic alliance, as well as a positive response to treatment that was associated with alliance, among abuse survivors compared to non-abused individuals. A meta-analysis of the general alliance literature found an alliance-outcome effect size of *r*=0.22 for a heterogeneous inpatient/outpatient population (Martin et al., 2000). In contrast, in a study of treatment outcome with women who had experienced CSA, Cloitre et al. (2004), found an alliance-outcome effect size of *r*=0.47. This moderate effect size among women who experienced CSA may indicate that alliance formation is even more influential a factor in treatment outcome among individuals with histories of interpersonal trauma. Given the degree of impairment and the length of treatment associated with DD, research about the association between alliance and treatment outcome in DD is sorely needed because of the possible role that alliance might have on these individuals’ response to treatment.

## Goals and hypotheses of the current study

Although experts in dissociation have advocated focusing on the therapeutic relationship as an important aspect of treating dissociative individuals, the treatment alliance has not been examined among individuals with DID/DDNOS. Since a strong therapeutic alliance positively impacts treatment outcome among general psychiatric patients, including trauma survivors, the next step would be to determine if the alliance is associated with positive outcome among DD. The current study aims to investigate the impact of alliance on treatment outcome using the international sample of DID/DDNOS patients and therapists who participated in the TOP DD study (Brand et al., 2009b, 2013). The findings may contribute to a better understanding of the alliance between DD patients and their therapists. Knowledge of how the alliance between DD patient and therapist relates to treatment outcome may be useful in improving treatment outcomes for this group.

The TOP DD researchers gathered data from therapists and patients regarding a range of variables, including symptoms, problematic behaviors, adaptive functioning, therapeutic alliance, and interventions, used in therapy sessions. We examined the relationship between patient- and therapist-rated alliance, and treatment outcome, specifically symptoms of dissociation, PTSD, and general psychiatric distress, as well as adaptive capacities that are expected to develop in treatment for DD (e.g., ability to tolerate emotion and maintain safety; degree of internal cooperation among dissociative states; ability to have healthy relationships). We hypothesized that a strong therapeutic alliance, as rated by both patients and therapists, would be associated with lower symptoms and higher adaptive capacities, after controlling for the adaptive functioning of the patient at the time when alliance data were collected.

## Methods

This study utilizes a subset of data from the TOP DD study (Brand et al., 2009b, 2013). The overall TOP DD study prospectively assessed patients four times across 30 months (Time 1–Time 4). However, this study only used data from Times 3 and 4 because the alliance measures were not included in the TOP DD study until those data collection points.

### Participants

Participants in the TOP DD study were comprised of patients and their therapists. The patient participants (*N*=132) used in these analyses were diagnosed with either DID or DDNOS; the methodology for the study has been described in detail earlier publications (Brand et al., 2009b, 2013). To be included in these analyses, patients had to have participated in the last two waves of TOP DD data collection. This widely diverse, international sample of therapists was recruited through various dissociation-related organizations and sources such as the member register of the International Society for the Study of Trauma and Dissociation (ISSTD), the ISSTD's Dissociative Disorders Psychotherapist Training Program, and online LISTSERV. Potential therapist participants were sent an email invitation to take part in the study. Therapists who were treating a patient whom they had diagnosed with DID or DDNOS were encouraged to invite that patient to participate in the study.

Due to high rates of comorbid disorders with DID and DDNOS, such as depression and PTSD, the authors sought to recruit a sample with limited exclusion criteria in order to collect a sample representative of the DD population. Thus, the only inclusion criteria were being 18 years of age or older and being able to read English (Brand et al., 2009b). Also, in order to be included in the study, therapists must have been “providing ongoing treatment of at least 3-month duration to one adult patient diagnosed with DID or DDNOS” (Brand et al., 2009b, p. 156).

During the course of the 30-month TOP DD study, many participants either dropped out, successfully ended treatment, or did not complete entire portions of all measures. Therefore, the sample sizes vary for each analysis presented in the current study.^1^

As is consistent with most DID/DDNOS research, this subsample was comprised of mostly women (roughly 96%), and the average age of patient participants who were in these analyses was 45.57 years. The age of patient participants ranged from 18 to 72 years. For a complete list of demographic data in the overall TOP DD sample, including race/ethnicity, abuse history, comorbid diagnoses, and countries of origin, see Brand et al. (2009b).

### Procedure

Upon agreeing to participate in the study, therapist participants were given a series of password-protected online surveys adapted from Zittel and Westen's (2005) survey of borderline personality disorder used in a similar naturalistic community study. To ensure confidentiality and to include patients without access to the Internet, all patient surveys were distributed to the therapists via postal mail; therapists distributed the surveys to the patients. Patients and therapists completed surveys at intake into the study, 6-, 18-, and 30-month intervals, although only Time 3 (18 month) and Time 4 (30 month) data are used in the current analyses.

### Predictor measures

#### Combined Alliance Short Form—Patient Version (CASF-P)

The CASF-P (Hatcher, 1999; Hatcher & Barends, 1996; Hatcher, Barends, Hansell, & Gutfreund, 1995) is a patient-rated measure of therapeutic alliance that combines three popular alliance measures: the California Psychotherapy Alliance Scales (Gaston & Marmar, 1994), the Working Alliance Inventory (Horvath & Greenberg, 1986) and the Helping Alliance Questionnaire (Luborsky, Johnson, & McLellan, 1994). It was developed through factor analyses of an outpatient community sample (*N*=231). This 20-item measure utilizes Likert scale responses, ranging from 1 (never) to 7 (always), to create four subscales, Confident Collaboration, Goals and Tasks, Bond, and Idealized Therapist. The CASF-P has demonstrated good reliability and validity, and in this sample, Cronbach's alpha was 0.93 at Times 3 and 4.

#### Working Alliance Inventory—Therapist Form (WAI-T)

Created from Bordin's tripartite conceptualization of the alliance, the WAI-T (Horvath & Greenberg, 1989; Hatcher, 1999) addresses the main themes of goal, task, and bond from the therapist's perspective. The version utilized in this study contains 24 items and is a composite score of four subscales identified by Hatcher (1999). The WAI-T is a widely used measure to indicate alliance and has good reliability and validity. In this sample, Cranach's alpha was 0.91 for Time 3 and 0.92 for Time 4.

### Outcome measures

#### Dissociative Experiences Scale (DES)

The DES (Bernstein & Putnam, 1986) measures the prevalence and severity of dissociative experiences. This 28-item self-report uses percentages (from 0% [never] to 100% [always]) to describe the frequency of varying degrees of dissociative experiences. A possible DD is indicated when the total score is 30 or above (Cardeña, 2008; Carlson, 1994). A meta-analysis of 26 studies by van Ijzendoorn and Schuengel (1996) found a test–retest reliability of 0.78 and total convergent validity between the DES and eight measures of dissociation of *r*=0.67. Cronbach's alpha was 0.90 for this patient sample.

#### Posttraumatic Stress Checklist—Civilian (PCL-C)

Created by Weathers, Litz, Huska, and Keane (1994), the PCL-C is a 17-item self-report measure that assesses the DSM-IV criteria for PTSD. Patients rate the severity of each symptom in terms of interference with daily functioning or distress, from 1 (not at all) to 5 (extremely). According to Weathers and Ford (1996), a score of 50 points or higher indicates the likely presence of PTSD. The PCL-C has been shown to have good to excellent reliability and validity. In this sample, Cronbach's alpha was 0.87.

#### Symptom Checklist-90-Revised (SCL-90-R)

Of the 90 self-report items that comprise this nine-subscale measure, the computed average of all scores, known as the Global Severity Index (GSI), is a reliable and valid indicator of overall symptomatology (Derogatis, 1994). The GSI has been shown to have good to excellent reliability and validity. Each item is rated on a scale of personal distress, from 0 (not at all) to 4 (extremely). In this sample, Cronbach's alpha for the GSI was 0.89.

#### Progress in Treatment Questionnaire (PITQ)

This therapist-rated questionnaire was created for the TOP DD study and is based on capacities described in the ISSTD's Guidelines for Treating Dissociative Identity Disorder in Adults (ISSTD, 2011; Brand et al., 2009b). The PITQ assesses the frequency with which DID/DDNOS patients demonstrate adaptive capacities that should develop including the ability to manage symptoms during staged, trauma-informed treatment for DD patients. Specifically, the PITQ measures the frequency with which the patient demonstrates abilities including “affect tolerance, impulse control, PTSD and dissociative symptom management skills, internal communication and cooperation among self states … and increasing ability to view self and others in an integrated, realistic way that is not dominated by trauma-based perceptions” (Brand et al., 2009b, p. 159). Therapists report what percentage of time (from 0 to 100%) the patient is capable of demonstrating each ability. Sample items include “The patient does not engage in potentially self-damaging acts such as abusing substances, purging, shoplifting, driving unsafely, or unsafe sex,” “The patient maintains personal physical safety (e.g., no cutting, burning or suicide attempts),” “The patient shows good affect tolerance,” and “The patient knows and uses containment strategies (e.g., hypnotic or imagery techniques used to contain intrusive PTSD symptoms) when they are needed.” The PITQ has good internal reliability (Cronbach's alpha=0.88 in the current patient subsample). In both the cross-sectional and longitudinal analyses from the larger TOP DD study, patients’ PITQ scores increased over time in treatment which coincided with improvements in GAF as well as reductions in symptoms (Brand et al., 2009b, 2013). Additional psychometric studies with the PITQ are currently underway.

## Results

Table 1 lists descriptive statistics for patient- and therapist-rated alliance and all outcome variables, measured at Times 3 and 4. As can be seen from the table, the variables showed a significant degree of variability and patients’ levels of functioning improved across the time periods of assessment, consistent with past research with this sample.

**Table 1:** Descriptive statistics of predictor and outcome variables

	Time 3				Time 4			
		
Variable	Mean	SD	N	Range	Mean	SD	N	Range
CASF-P	5.97	0.82	148	2.05–7.00				
WAI-T	5.84	0.61	169	3.38–7.00	5.83	0.62	145	3.50–7.00
DES	29.57	21.17	135	1.79–95.71	28.47	20.12	131	1.07–91.43
PCL-C	51.94	15.79	135	20.00–85.00	50.40	15.29	131	20.00–80.00
GSI	1.63	0.78	134	0.20–3.59	1.58	0.83	131	0.02–3.44
PITQ	212.20	49.26	169	89.00–314.00	211.33	52.08	145	80.00–315.00

CASF-P=Combined Alliance Short Form—Patient Version; WAI-T=Working Alliance Inventory—Therapist Form; DES=Dissociative Experiences Scale-II; PCL-C=Posttraumatic Checklist—Civilian; GSI=Global Severity Index (Symptom-Checklist 90—Revised); PITQ=Progress in Treatment Questionnaire.

Table 2 presents correlations between patient and therapist-rated alliance at Time 3 and all Time 3 and Time 4 outcome measures. We found that patient and therapist ratings of the alliance were moderately intercorrelated, supporting the construct validity of these measures but also suggesting that there is substantial variability in how the alliance is viewed from the therapist versus the client's point-of-view. Turning to the main hypotheses of this study, we found that both patient-rated and therapist-rated alliance were significantly associated with fewer symptoms and better overall functioning, both cross-sectionally at Time 3, and longitudinally, from Time 3 alliance ratings to Time 4 outcomes. Effect sizes were in the moderate-to-large range for many of these correlations. Although these correlational relationships suggest that the alliance is an important correlate of better outcomes in this population, it is important to determine whether the alliance predicted better outcomes, even after controlling for the patients’ ability to manage their symptoms at Time 3. We turn to those analyses next.

**Table 2:** Correlations between patient-rated alliance, therapist-rated alliance, and Time 3 and Time 4 outcomes

Measure	1	2	3	4	5	6	7	8	9	10	11
1. CASF-P (Time 3)	–										
2. WAI-T (Time 3)	0.28	–									
3. WAI-T (Time 4)	0.42	0.68	–								
4. DES (Time 3)	−0.39	−0.16	−0.22	–							
5. PCLC (Time 3)	−0.46	−0.20	−0.30	0.73	–						
6. GSI (Time 3)	−0.44	−0.24	−0.36	0.69	0.85	–					
7. PITQ (Time 3)	0.28	0.60	0.60	−0.31	−0.40	−0.42	–				
8. DES (Time 4)	−0.43	−0.22	−0.28	0.81	0.66	0.63	−0.39	–			
9. PCLC (Time 4)	−0.45	−0.28	−0.43	0.68	0.77	0.74	−0.48	0.80	–		
10. GSI (Time 4)	−0.42	−0.24	−0.39	0.62	0.75	0.81	−0.45	0.76	0.87	–	
11. PITQ (Time 4)	0.31	0.43	0.68	−0.38	−0.46	−0.51	0.78	−0.41	−0.53	−0.52	–

Critical values for *p<*0.05 and *p<*0.01 are *r*=0.178 and 0.232, respectively, for df=120.

CASF-P=Combined Alliance Short Form—Patient Version; WAI-T=Working Alliance Inventory—Therapist Form; DES=Dissociative Experiences Scale-II; PCL-C=Posttraumatic Checklist—Civilian; GSI=Global Severity Index (Symptom-Checklist 90—Revised); PITQ=Progress in Treatment Questionnaire.

We computed a series of regression equations, in which progress in treatment (PITQ) scores at Time 3 were entered in step 1 to control for ability to manage symptoms followed by patient-rated therapeutic alliance at Time 3, to predict each of our four outcome variables, as assessed at Time 4. To predict PITQ as an outcome variable, we used GSI at Time 3 as a covariate. As seen in Table 3, for the DES, PCL-C, and GSI, the alliance accounted for unique variability in the prediction of Time 4 functioning, after controlling for the patient's progress in treatment at Time 3. The amount of unique variance accounted for by the alliance ranged from 7 to 11% when covarying PITQ scores. However, when covarying GSI scores, patient-rated alliance did not predict PITQ outcome scores at Time 4. When we repeated these analyses using therapist-rated alliance at Time 3 we found that the alliance predicted better PITQ ratings at Time 4 after controlling for overall symptoms with the GSI (R^2^ change=0.07, *p*=0.003). Therapist-rated alliance did not uniquely predict any of the other outcome measures.

**Table 3:** Regression analyses of patient-rated alliance (Time 3) predicting Time 4 outcomes when controlling for progress in therapy or global functioning (Time 3)

Variables entered and dependent variables	B	SE B	β	R^2^	ΔR^2^
DES—Time 4					
Step 1				0.17**	
PITQ (Time 3)	−0.18	0.04	−0.41**		
Step 2				0.25**	0.08**
CASF-P	−7.89	2.59	−0.29**		
PCL-C—Time 4					
Step 1				0.25**	
PITQ (Time 3)	−0.17	0.03	−0.50**		
Step 2				0.36**	0.11**
CASF-P	−6.85	1.78	−0.34**		
GSI—Time 4					
Step 1				0.25**	
PITQ (Time 3)	−0.01	0.00	−0.50**		
Step 2				0.32**	0.07**
CASF-P	−0.29	0.10	−0.27**		
PITQ—Time 4					
Step 1				0.26**	
GSI (Time 3)	−30.01	5.21	−0.51**		
Step 2				0.27	0.01
CASF-P	7.91	6.00	0.13		

** *p<*0.01; *p<0.05.

CASF-P=Combined Alliance Short Form—Patient Version; WAI-T=Working Alliance Inventory—Therapist Form; DES=Dissociative Experiences Scale-II; PCL-C=Posttraumatic Checklist—Civilian; GSI=Global Severity Index (Symptom-Checklist 90—Revised); PITQ=Progress in Treatment Questionnaire.

## Discussion

The goal of this study was to assess whether patient- and therapist-rated alliance is associated with improvements in symptoms and functioning for individuals with DID or DDNOS. This investigation reveals some promising insights into treatment outcome for this challenging population. Consistent with previous literature on alliance formation in traumatized patients, the patients in this data sample were able to successfully form a working alliance in the majority of cases. Patients’ and therapists’ ratings of alliance were in the range found among traumatized, non-DID/DDNOS samples (Price et al., 2004). Additionally, in accordance with the literature, patient and therapist-rated alliance scores were moderately to strongly correlated with lower levels of dissociation, PTSD symptoms, and general distress, as well as greater adaptive capacities in both cross-sectional and longitudinal analyses.

Moreover, we found that when controlling for adaptive capacities, including ability to manage symptoms, patients’ ratings of alliance predicted improvement in functioning over time across a wider range of outcomes than did therapists’ ratings of the alliance. Specifically, we found that whereas therapist-rated alliance at Time 3 was only significantly associated with therapist-rated adaptive capacities at Time 4,^2^ patient-rated alliance scores were significantly associated with better patient-rated outcome measures across all three symptom domains (i.e., DES, PCL-C, and GSI). Consistent with prior findings (Martin et al., 2000), one may infer that patients may be better at accurately indicating the strength of the alliance than therapists, or that their view of the alliance is more predictive of improvement in symptoms. This is particularly interesting for DD patients, as some writers have commented that dissociative individuals are supposedly fantasy-prone and suggestible, and that they are unable to provide credible subjective opinions and self-report data (Giesbrecht, Lynn, Lilienfeld, & Merckelbach, 2008). This “fantasy model” of dissociation has been recently thoroughly reviewed and discredited (Dalenberg et al., 2012). The current study provides further evidence that dissociative patients’ assessments of relationships are valid and have predictive value over time.

The working alliance has been consistently reported as an important factor in the therapeutic process and has been studied since the time of Freud's career (Freud, 1958; Bordin, 1979; Ackerman & Hilsenroth, 2003; Hilsenroth et al., 2004). Most of the clients in the TOP DD study scored high on the patient-rated alliance measure utilized in the study compared to other studies, suggesting they are capable of forming collaborative bonds with others (Keller et al., 2010; Price et al., 2004). DD patients almost always report histories of severe trauma, often by attachment figures or adults in charge of providing care to them (Brand et al., 2009b). It is a personality strength that many of these individuals can form therapeutic bonds with their therapists, despite chronic interpersonal trauma during the developmental years. Another study found that DID patients are often capable of seeing others as potentially collaborative despite also being vulnerable to trauma-based fears of being hurt or manipulated by others. DID patients were found to have higher scores indicative of cooperative perceptions of others on the Rorschach than did patients with psychotic spectrum disorders or borderline personality disorder (Brand et al., 2009a).

Despite potential initial difficulties in forming an alliance, traumatized individuals who create strong alliances in therapy have healthier treatment outcomes than those without strong alliances (Paivio & Bahr, 1998; Paivio & Patterson, 1999). In the current study, alliance was measured only at Time 3 and Time 4. Patients who may have had initial difficulties in forming alliances at earlier times may have been able to achieve a strong alliance by the time it was measured. Due to its relative stability over treatment time (Hilsenroth et al., 2004), this is not considered a major problem or limitation. However, it is also possible that patients who had difficulty forming a solid alliance may have dropped out of treatment or not been willing to participate in a research study.

Individual studies and meta-analyses show a consistent relationship between alliance strength and therapeutic outcome (Paivio & Bahr, 1998; Paivio & Patterson, 1999; Martin et al., 2000). The patient reported r-effect sizes in this study (0.28–0.46) are consistent with, and at times higher than, the average of 0.2–0.3 that has been found in prior meta-analyses (Martin et al., 2000). Thus, the data suggests that DD patients who have a shared sense of goals, tasks, and trust with their therapists and can speak with them about the relationship itself, can utilize these skills and emotions to assist them in their recovery. Social support is a key factor in trauma recovery, and has been shown to contribute to the development of an early positive alliance (Keller et al., 2010). Given our finding about the strength of the relationship between alliance and outcome, a strong relationship with the therapist appears to be a crucial relationship in the dissociative individual's life. Trauma experts have emphasized the importance of the relationship in treating individuals who have experienced severe, chronic trauma, including dissociative individuals (Courtois & Ford, 2013; Dalenberg, 2004; Herman, 1997; Kluft, 1993a, 1993b; Kluft & Loewenstein, 2007; Loewenstein, 1993). These findings support those recommendations. The ability to effectively work with a therapist and collaborate together towards achieving the goal of improved functioning may allow the patient to more deeply engage in the therapeutic process, address and resolve trauma-based beliefs about relationships, and ultimately display fewer symptoms and better adaptive capacities.

A criticism of alliance-outcome research is the potential for confounding effects of symptom improvement on alliance scores. A recently published study attempted to address this problem by assessing within-patient improvement and its effects on alliance. Falkenström, Granström, and Holmqvist (2013) found that while symptom improvement does contribute to higher alliance scores among some patients, it is not the sole cause or indicator of the alliance itself. Within the current study, we controlled for therapist-rated progress in treatment which includes the ability to manage symptoms as a means of preventing the confounding of symptoms with alliance scores.

The results of this study must be interpreted with consideration of its methodological and statistical limitations. Because the TOP DD study is a naturalistic study, we can conclude that stronger alliance is related to fewer symptoms across all the measures we analyzed, although we cannot make conclusions about causality. Additionally, we do not have alliance data over the full course of the TOP DD study or from the beginning of treatment. There are additional variables that might have affected alliance earlier in the treatment. Due to successful completion of treatment as well as drop-out from the study, we could use only a subset of the participants who originally enrolled in the study. These results may not generalize to all DD patients. For example, there may be DD patients who are so severely damaged by interpersonal trauma that they do not seek therapy, they drop out prematurely, or they do not participate in research; these individuals may be less able to develop an alliance than the participants in this study. Further studies should investigate alliance and its association with outcome from the beginning of treatment, and attempt to keep the drop-out rate low.

In summary, the strength of alliance scores and their association with therapeutic outcome is a significant advance in understanding what seems to contribute to DD patients responding well in therapy. The findings of this study provide a substantial first look into therapeutic bonds in DD patients. These patients can form alliances as high in strength as can other patient groups. Consistent with findings that show alliance as a key factor in therapeutic progress, this study found that the alliance is a crucial variable in treatment outcome for DD patients. Therapists who work with DD patients should emphasize the importance of the alliance, perhaps particularly due to the patient's trauma history or relationship problems. These findings have implications for training clinicians so they can be successful in working with DID clients; clinicians must learn to develop a strong alliance with these patients. Future research to clarify processes that help develop and maintain a strong alliance with DD patients would be useful.

The success of the TOP DD study will hopefully encourage other researchers to conduct similar studies using naturalistic designs with DD patients. Such studies are needed to examine additional variables that increase or decrease the likelihood of therapeutic success and improvement in DD patients including the degree of social support outside of the therapy relationship, the skill level of the clinician, and the types of interventions used. In conclusion, the working alliance is a key factor in the improvement of symptoms and overall adaptive capacities in DD patients. Further research on the alliance and other variables that influence therapeutic outcome in DD patients is strongly indicated.

## Footnotes

1 Sample sizes for each analysis are as follows: DES Time 3 and Time 4: *n*=134 and *n*=113, respectively PCL-C Time 3 and Time 4: *n*=134 and *n*=113, respectively GSI Time 3 and Time 4: *n*=133 and *n*=113, respectively PITQ Time 3 and Time 4: *n*=119 and *n*=110, respectively.

## Footnotes

2 These analyses were repeated using the California Psychotherapy Alliance Scales (CALPAS), another therapist-rated alliance measure, and revealed near-identical results.
